# Taxonomy and identification of bacteria associated with acute oak decline

**DOI:** 10.1007/s11274-017-2296-4

**Published:** 2017-06-16

**Authors:** Carrie Brady, Dawn Arnold, James McDonald, Sandra Denman

**Affiliations:** 1 Faculty of Health and Life Sciences, Centre for Research in Bioscience, University of the West of England, Frenchay Campus, Coldharbour Lane, Bristol, BS16 1QY UK; 2 School of Biological Sciences, Bangor University, Bangor, Gwynedd UK; 3 Centre for Ecosystems, Society and Biosecurity, Forest Research, Alice Holt Lodge, Surrey, UK

**Keywords:** Acute oak decline, *Brenneria goodwinii*, *Gibbsiella quercinecans*, Taxonomy

## Abstract

Acute oak decline (AOD) is a relatively newly described disorder affecting native oak species in Britain. Symptomatic trees are characterised by stem bleeds from vertical fissures, necrotic lesions in the live tissue beneath and larval galleries of the two spotted oak buprestid (*Agrilus biguttatus*). Several abiotic and biotic factors can be responsible for tree death, however the tissue necrosis and stem weeping is thought to be caused by a combination of bacterial species. Following investigations of the current episode of AOD which began in 2008, numerous strains belonging to several different bacteria in the family *Enterobacteriaceae* have been consistently isolated from symptomatic tissue. The majority of these enterobacteria were found to be novel species, subspecies and even genera, which have now been formally classified. The most frequently isolated species from symptomatic oak are *Gibbsiella quercinecans, Brenneria goodwinii* and *Rahnella victoriana*. Identification of these bacteria is difficult due to similarities in colony morphology, phenotypic profile and 16S rRNA gene sequences. Current identification relies heavily on *gyrB* gene amplification and sequencing, which is time consuming and laborious. However, newer techniques based on detection of single nucleotide polymorphisms show greater promise for rapid and reliable identification of the bacteria associated with AOD.

## Introduction

Acute oak decline (AOD) is a relatively newly described syndrome in Great Britain (Denman and Webber 2009), which mostly affects mature oak trees of the native species *Quercus robur* (pedunculate oak) and *Quercus petraea* (sessile oak), and to a lesser extent, the non-native species *Quercus cerris* (turkey oak). Reports of oak in Britain suffering from dieback and decline date back over 100 years (Delatour 1983; Gibbs and Greig 1997). However, the current episode of AOD appears to cause a more rapid decline with severely affected trees dying in 4–5 years after symptoms are noted (Denman et al. 2010). Whereas previous episodes of AOD mainly affected the tree foliage and key causal agents were identified as the insect defoliato (*Totrix viridana*) and powdery mildew (*Erysiphe alphitoides*) (Day 1927; Robinson 1927; Osmaston 1927), the current outbreak of AOD causes damage to the inner bark and trunk of the tree, and a native beetle, *Agrilus biguttatus* and bacterial species are implicated in causal roles.

**Fig. 1:**
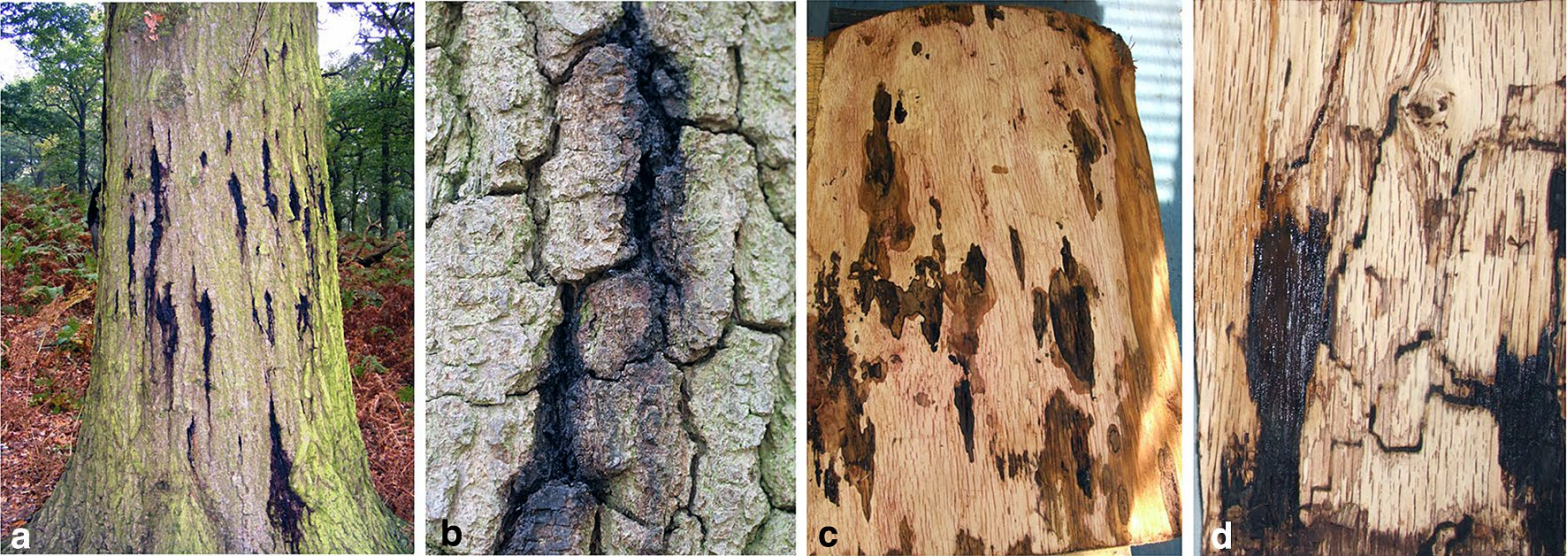
Symptoms of acute oak decline **a** external symptoms of weeping stem bleeds **b** cracked bark plates caused by necrotic underlying tissue **c** lesions in the inner bark **d** *Agrilus biguttatus* larval galleries in close proximity to necrotic lesions

The first external evidence of symptomatic oak suffering from the current AOD outbreak, is stem bleeding. This typically occurs in spring (March–June) and autumn (October–November), but there is some variation (Denman et al. 2014). The stem bleeds appear as weeping patches of clear dark exudate (Fig. 1a) and can be situated as close as 5 cm together to 22 cm apart, in both the vertical and horizontal directions. In more advanced cases, cracks of 3–15 cm can develop between the bark plates possibly when the underlying tissue has decayed (Fig. 1b). When the outer bark is removed, lesions are present in the inner bark as dark, irregularly shaped wet patches (Fig. 1c). These can extend down to the sapwood but do not enter the heartwood. Almost always, galleries of the two spotted oak borer (TSOB), *Agrilus biguttatus*, are found in close proximity to the lesions in the phloem and sapwood, indicating signs of larval activity (Fig. 1d). In summary, there are four key symptoms which are indicative of AOD: (1) weeping patches on oak trunks, (2) dark fluid seeping from cracks in the outer bark, (3) irregular oval-shaped lesions in inner bark and/or cavities behind the outer bark and (4) presence of larval galleries of TSOB close to the lesions (Denman et al. 2014).

**Fig. 2:**
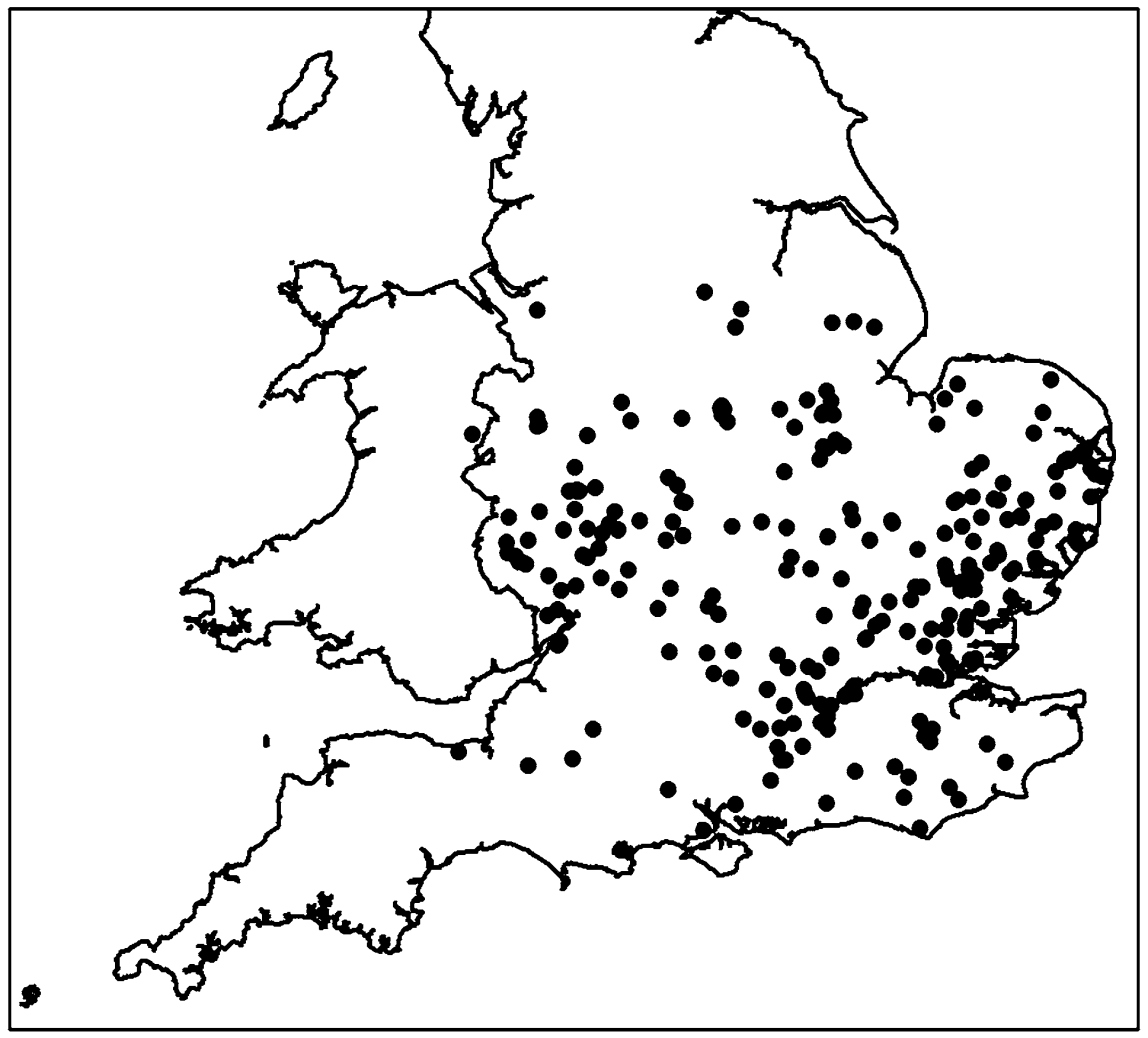
Current distribution of acute oak decline in Britain (all sites verified as of 2014)

Symptoms of the current episode of AOD in Britain were first noted in east England in the 1980s and in the years following, consistently increasing reports of decline symptoms were made to the Tree Health Diagnostic and Advisory Service of Forest Research in Britain (http://www.forestry.gov.uk/fr/INFD-5UWEY6), allowing the known distribution of AOD to be mapped. It appears that AOD has spread from East Anglia (the south east of England) to the midlands bordering Wales, with the majority of cases noted in the south east. The currently available distribution map (upto 2014) shows that AOD affected sites now extend to the midlands and south west of England and further west into Wales (Fig. 2). The highest incidence of AOD still occurs in the south east of England.

Both biotic and abiotic factors appear to contribute to the rapid decline of oak suffering from AOD. It is thought that trees are predisposed to AOD due to local biotic causes as well as environmental factors (Brown et al. 2016). Several bacterial species are consistently isolated from the lesions, necrotic tissue and exudate of symptomatic oak indicating that they play a key role in development of AOD. However, little is known about the epidemiology of AOD, the transmission of the bacteria or the precise sequence of events which lead to the symptoms observed. TSOB is also acknowledged to contribute to the decline of symptomatic oak. A previous study on the role of *A. biguttatus* in declining European oak in Belgium, indicated that the buprestid colonises the oak tissue before the external symptoms of stem bleeding and cracked bark plates are observed (Vansteenkiste et al. 2004). In Britain, stem bleeds on oak have been observed and recorded before the appearance of the D-shaped exit holes of the adult *A. biguttatus* (Brown 2014), implying that colonisation of oak stems does not necessarily occur first.

The majority of the bacteria isolated from symptomatic oak were found to belong to novel species or genera within the family *Enterobacteriaceae* (Brady et al. 2010b, 2012, 2014a, b; Denman et al. 2012). Additionally, a large number of *Pseudomonadaecae* were isolated (Denman et al. 2016). It was clear at the start of these investigations that more than one bacterial species could play a role in symptom development. However, before their roles could be elucidated, it was necessary to formally identify and classify the bacteria which didn’t belong to any existing genus or species.

### Taxonomy

The first bacterial isolates from trees suffering from symptoms of AOD were isolated over two seasons from 2008 to 2009 at 13 different parks and woodlands in Britain (Brady et al. 2010b). The results from Gram staining, KOH and oxidation/fermentation tests indicated that the majority of these bacteria were Gram-negative and facultatively anaerobic, placing them within the family *Enterobacteriaceae*. Despite the frequent inability of 16S rRNA gene sequencing to reliably identify members of this family, the almost complete gene sequences were determined for a selection of strains. Based on the findings of Biosca et al. (2003) who suggested *Brenneria quercina* was responsible for a similar decline observed on Mediterranean oak (*Q. ilex* and *Q. pyrenaica*) in Spain, it was expected that the 16S rRNA gene sequences for the AOD isolates from Britain would show highest similarity to species of the genus *Brenneria*. Instead the majority of the isolates showed highest similarity to species of *Serratia* and other *Enterobacteriaceae* such as *Kluyvera, Klebsiella* and *Raoultella* (Brady et al. 2010b). Two smaller groups of isolates showed highest 16S rRNA gene similarity to species of *Brenneria* (Brady et al. 2012; Denman et al. 2012). This was more in keeping with the results from a later study on the Mediterranean oak isolates, where some isolates were shown to belong to *Brenneria*, but another group of isolates were closely related to *Serratia* species (Poza-Carrión et al. 2008). A phylogenetic tree based on 16S rRNA gene sequences placed the British ‘*Serratia*-like’ isolates and two Spanish isolates from the Poza-Carrión et al. (2008) study in a single cluster with *Serratia rubidaea* as the closest phylogenetic neighbour. The ‘*Brenneria*-like’ isolates were divided into two main clusters, one with *Brenneria salicis* (the type species) as the closest phylogenetic neighbour and the other most closely related to *Brenneria quercina*, which was far removed from the remaining *Brenneria* species. Additionally, the three groups of isolates differed in their colony and cell morphology and their motile ability (Brady et al. 2010b, 2012; Denman et al. 2012).

In an attempt to further classify the largest group of isolates, those showing highest similarity to *Serratia* species, protein-encoding gene sequences were investigated as they were known to display more heterogeneity between closely related enterobacteria. Sequences of species of *Serratia, Klebsiella, Enterobacter, Kluyvera, Raoultella* and *Edwardsiella* were available from GenBank for both DNA gyrase (*gyrB*) and RNA polymerase β subunit (*rpoB*) and showed high resolving power for these genera. The two genes were sequenced for 18 of the ‘*Serratia*-like’ oak isolates from Britain and two oak isolates from Spain. Maximum likelihood phylogenetic trees were constructed using the relevant evolutionary models. In both the *gyrB* and *rpoB* phylogenies, the isolates were contained in a single well-supported cluster which wasn’t included in either the *Serratia* or *Edwardsiella* clades (the closest phylogenetic relatives). Both of these phylogenies suggested that the ‘*Serratia*-like’ isolates actually belonged to a single species within a novel genus. DNA–DNA hybridization values confirmed that the isolates belonged to a single species, with five representative strains demonstrating 78–93% similarity to each other. Phenotypically, the isolates could be distinguished from species of *Serratia* and *Edwardsiella* thereby fulfilling the criteria for a novel genus and species. As such, this first group of isolates was formally described and classified as *Gibbsiella quercinecans* gen. nov., sp. nov (Brady et al. 2010b). Strains belonging to this genus and species are Gram-negative short rods with no flagella. On nutrient agar, the colonies are white to cream, round, convex and smooth with entire margins.

The two groups of ‘*Brenneria*-like’ isolates proved more difficult to classify due to the polyphyletic nature of the genus *Brenneria*. Not unusually, when 16S rRNA gene sequences of *Brenneria* species were examined in a phylogenetic tree, they failed to form a monophyletic clade. This is not unusual or rare for members of the *Enterobacteriaceae*. Indeed genera such as *Erwinia* and *Pantoea* appear polyphyletic based on their 16S rRNA gene phylogeny. However, these genera both form monophyletic clades when their phylogeny is based on protein-encoding genes (Moretti et al. 2011; Brady et al. 2010a), whereas *Brenneria* did not. Phylogenetic trees based on *gyrB* sequences further demonstrated the polyphyletic nature of *Brenneria*, with four species of *Brenneria* (*B. salicis, B. alni, B. nigrifluens* and *B. rubrifaciens*) contained in a single clade and *B. quercina* far removed and situated on a separate branch. Furthermore, oak isolates from both Britain and Spain clustered within the *B. quercina* clade indicating that in order to classify these strains, some taxonomic rearrangements would have to be made in the genus *Brenneria* (unpublished data).

Around the same time, a large taxonomic study was in preparation to examine the phylogeny of all plant-pathogenic and plant-associated genera within the family *Enterobacteriaceae* using a combination of protein-encoding genes in a multilocus sequence analysis (MLSA) scheme. The term MLSA was coined in 2005 to refer to phylogenetic analyses based on the partial nucleotide sequences of several protein-encoding genes (Gevers et al. 2005). An MLSA scheme, initially developed to examine the taxonomy of *Pantoea* species, using short stretches of the DNA gyrase (*gyrB*), RNA polymerase β subunit (*rpoB*), ATP synthase β subunit (*atpD*) and initiation translation factor 2 (*infB*) was found to be particularly effective at discovering true phylogenetic relationships (Brady et al. 2008). As the ‘*B. quercina*-like’ oak isolates from Britain and Spain would impact any phylogenetic analyses of the plant-pathogenic and plant-associated genera of the *Enterobacteriaceae*, it was decided to include them in the study along with all species belonging to *Brenneria, Dickeya, Erwinia* and *Pectobacterium*.

**Fig. 3:**
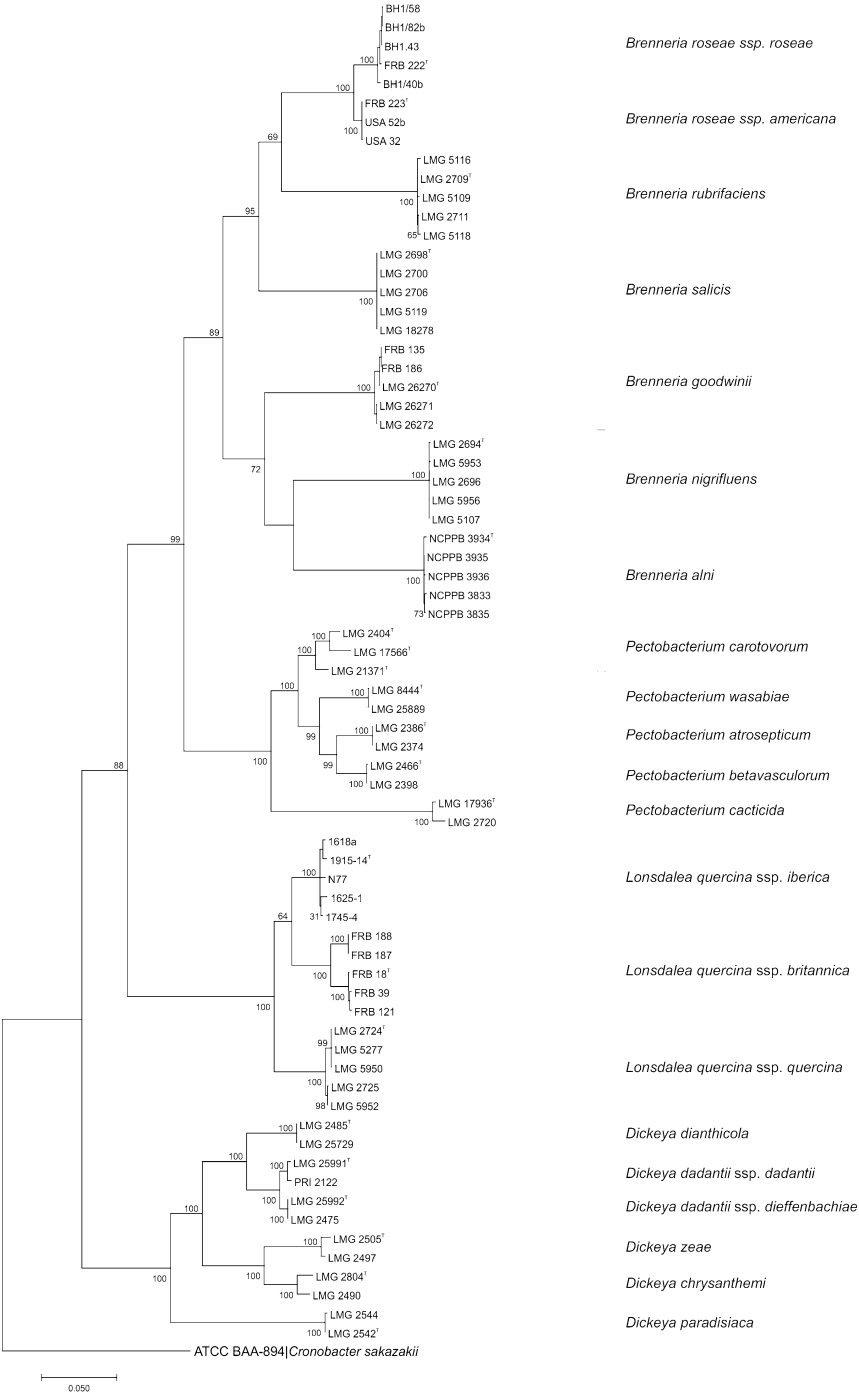
Maximum likelihood tree based on concatenated partial gene sequences of *gyrB, rpoB, atpD* and *infB* of members of the genera *Brenneria, Lonsdalea* phylogenetically related species. The phylogenetic analysis was inferred using the general time reversible model with estimated gamma distribution with invariant sites (G + I) in MEGA 7 (Kumar et al. 2016). Bootstrap values after 500 replicates are expressed as percentages. *Cronobacter sakazakii* is included as an outgroup; gene sequences were obtained from the genome sequencing database (http://www.ncbi.nlm.nih.gov). *Bar* 0.05 substitutions per site

Concatenated analysis of the four genes revealed that *B. quercina* reference strains, along with the ‘*B. quercina*-like’ oak isolates from Britain and Spain formed a stable highly supported clade distinctly separate from the genus *Brenneria*. The distance of the clade from the remaining *Brenneria* species, which were contained in their own strongly supported clade, indicated that *B. quercina*, along with the oak isolates from Britain and Spain constituted a novel genus (Fig. 3). Within this novel genus, three clusters could be delineated. The reference strains of *B. quercina* formed one group, while the oak isolates from *Q. robur* in Britain were contained in the second cluster and the Spanish isolates from Mediterranean oak were contained in the third. The clear differentiation of these three groups and their 100% bootstrap support suggested that each could be considered a separate taxon. However, the DNA–DNA hybridization values between these three taxa were considered to be borderline ranging from 51 to 73%, when similarity values over 70% typically delineate different species (Wayne et al. 1987). Due to their similar colony morphology and their association with oak species, it was proposed to classify the three taxa as subspecies of a single species within a novel genus. *B. quercina* was transferred to the novel genus *Lonsdalea*, as *L. quercina* ssp. *quercina* ssp. nov., the oak isolates Britain were classified as *L. quercina* ssp. *britannica* ssp. nov. while the oak isolates from Spain were classified as *L. quercina* ssp. *iberica* ssp. nov. (Brady et al. 2012). Strains belonging to this species or subspecies are Gram-negative short rods and are motile by peritrichous flagella. On tryptone soya agar colonies are white to cream, small and round, convex and smooth with entire margins, they appear almost watery and are difficult to pick up with an inoculation loop. They also grow at a slower rate than *G. quercinecans* in both solid and liquid media.

Once the taxonomic rearrangements within *Brenneria* were finalised by the transfer of *B. quercina* to the genus *Lonsdalea*, the formal description of the third group of initial oak isolates from Britain (those which clustered within the genus *Brenneria*) was relatively straightforward. Phylogenetic analysis of the MLSA sequences of a selection of these isolates placed them firmly within the genus *Brenneria* in a single distinct cluster with 100% bootstrap support (Fig. 3). DNA–DNA hybridization confirmed that they constituted a single taxon and phenotypically, the isolates could be differentiated from the other species in the genus *Brenneria*. The name *B. goodwinii* sp. nov. was proposed for this group of oak isolates from Britain (Denman et al. 2012). Strains belonging to this species are Gram-negative short rods and motile by peritrichous flagella. On nutrient agar, colonies are pale cream, round, convex and smooth with entire margins. Like *L. quercina*, cultures grow at a slower rate compared to *G. quercinecans* in both solid and liquid media.

While the taxonomic rearrangements and novel genus and species descriptions were being made to classify the first bacterial isolates from symptomatic oak, ongoing isolations were taking place from new sites of the decline. Using *gyrB* gene sequencing (or the full MLSA scheme if necessary), most of these isolates could be assigned to *G. quercinecans, B. goodwinii* or *L. quercina* ssp. *britannica*. However, it was found that a number of isolates from symptomatic oak, alder and walnut log samples (from pathogenicity trials of *Brenneria* species), and buprestid beetles between 2009 and 2012 could not be assigned to any existing species within the family *Enterobacteriaceae* (Brady et al. 2014a, b). These isolates could be divided into two main groups, those which showed highest *gyrB* sequence similarity to *Brenneria* species and those which were most similar to *Rahnella aquatilis*.

The isolates appearing to belong to yet another novel species within *Brenneria* were isolated from symptomatic oak in both Britain and the USA, although in much lower numbers than the frequently isolated *B. goodwinii*. Following phylogenetic analysis based on the MLSA scheme, it was observed that the isolates formed two well-supported and closely related clusters relating to their country of origin (Fig. 3). The phylogenetic distance between the two clusters was closer than that usually observed between different species of *Brenneria*, suggesting that the isolates could belong to two subspecies of a novel species. An alternative measure of the whole DNA relatedness of the isolates using a fluorometric real time PCR method was implemented instead of the usual DNA–DNA hybridization method. The Δ*T*_m_ (corresponding to the mean difference between the *T*_m_ of the hybrid and reference DNA) was determined between strains from both Britain and the USA. Δ*T*_m_ values of less than 5.2 °C were observed amongst the isolates from Britain and the USA, confirming that the isolates from both clusters constituted a single taxon. Currently, a Δ*T*_m_ value of 5 °C is the accepted limit for species circumscription within bacteria (Rosselló-Móra and Amann 2001). Values significantly above 5 °C are an indication of different species, as in the case of strains from both clusters and *B. goodwinii* demonstrating Δ*T*_m_ values of 7.2–9.2 °C. Although the DNA–DNA relatedness between the isolates from Britain and the USA indicated that they belonged to a single taxon, there was sufficient evidence from the MLSA phylogenies, further genotypic data and several phenotypic characteristics that warranted their classification as two subspecies within a novel species. The names *Brenneria roseae* ssp. *roseae* and *Brenneria roseae* ssp. *americana* were proposed for the isolates from Britain and the USA, respectively (Brady et al. 2014a). Strains belonging to *B. roseae* are Gram-negative rods which are motile by peritrichous flagella. Colonies are pale cream, round, convex and smooth with entire margins on nutrient agar.

**Fig. 4:**
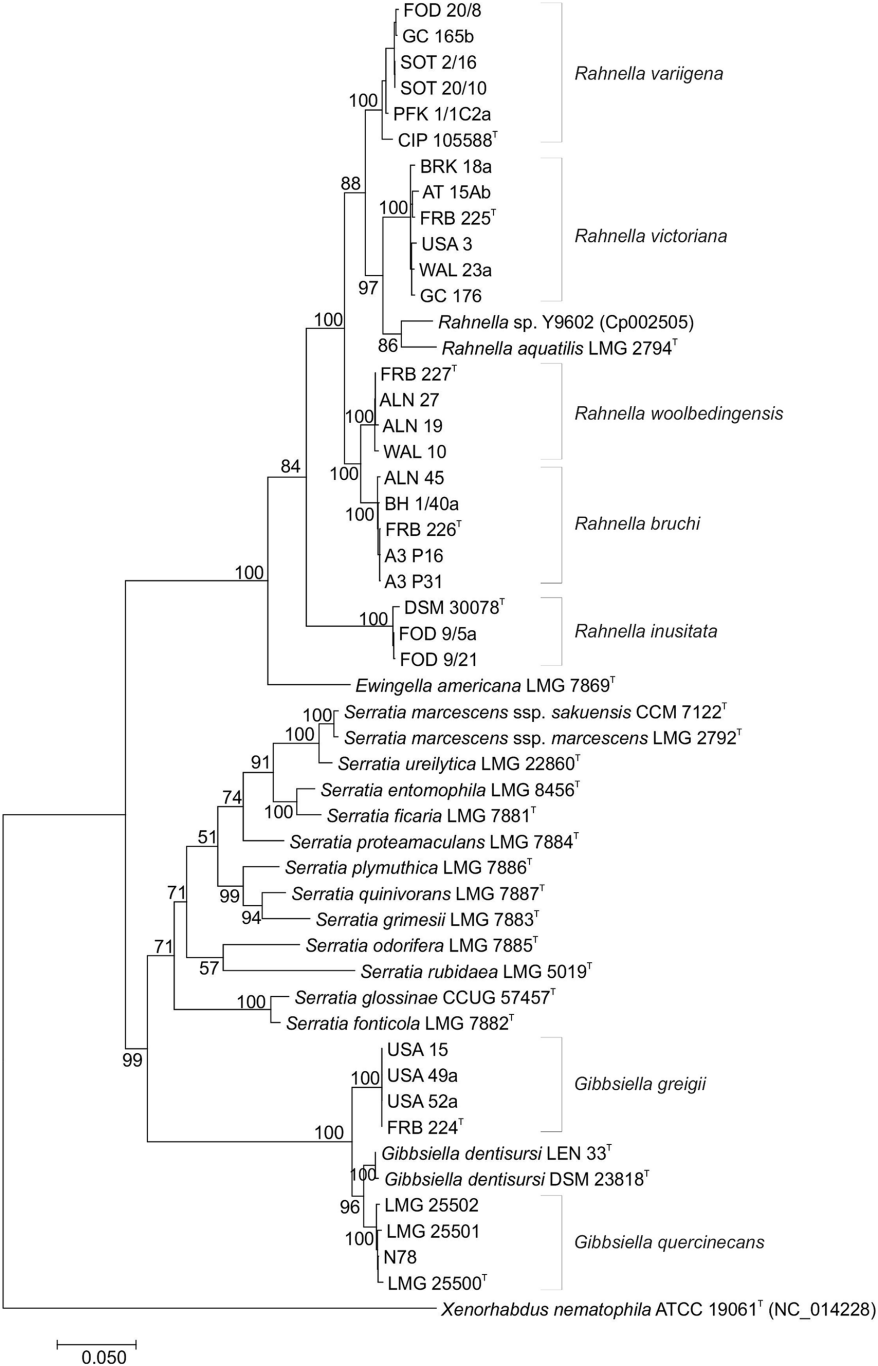
Maximum likelihood tree based on concatenated partial gene sequences of *gyrB, rpoB, atpD* and *infB* of members of the genera *Rahnella, Gibbsiella* and phylogenetically related species. The phylogenetic analysis was inferred using the General Time Reversible model with estimated gamma distribution with invariant sites (G + I) in MEGA 7 (Kumar et al. 2016). Bootstrap values after 500 replicates are expressed as percentages. *Xenorhabdus nematophila* is included as an outgroup; gene sequences were obtained from the genome sequencing database (http://www.ncbi.nlm.nih.gov). *Bar* 0.05 substitutions per site

The group of isolates showing highest similarity to *R. aquatilis* were isolated from symptomatic oak in Britain and the USA, alder and walnut log tissue and *A. biguttatus*; and are the most recent novel species to be described for bacteria associated with acute oak decline. The genus *Rahnella* originally consisted of only one species, *R. aquatilis*, which has been found to be widely distributed in the environment, and two genomospecies (a species which can only be characterised by its DNA and cannot be phenotypically differentiated from related species). MLSA of *R. aquatilis*, the unclassified genomospecies 1 and 2 and the diverse group of isolates revealed the presence of five MLSA groups (strongly supported clusters, each equivalent to a single taxon) within *Rahnella*, two of which corresponded with the unclassified genomospecies (Fig. 4). DNA–DNA relatedness studies, using both the fluorometric real time PCR method and DNA–DNA hybridization, confirmed the status of each taxon as a single novel species. All five novel species could be differentiated phenotypically and genotypically, from each other and from *R. aquatilis* (Brady et al. 2014b). The largest MLSA group (MLSA group 1) contained mostly oak isolates from Britain and the USA was named *Rahnella victoriana* sp. nov., while MLSA group 2 (named *Rahnella variigena* sp. nov.) consisted of oak isolates from only Britain and the reference strain for genomospecies 2. The reference strain of genomospecies 3 and oak isolates from Britain constituted MLSA group 3 or *Rahnella inusitata* sp. nov., and MLSA group 4 named as *Rahnella bruchi* sp. nov. contained only strains isolated from the gut of *A. biguttatus*. Isolates from alder and walnut tissue clustered in MLSA group 5 which was named *Rahnella woolbedingensis* sp. nov. All five novel *Rahnella* species are Gram-negative rods of varying lengths, and are motile. On nutrient agar colonies are cream, round, convex and smooth with entire margins. Cultures grow at a faster rate than *Brenneria* and *Lonsdalea* species in both solid and liquid media.

The diverse origins of the isolates suggested that these *Rahnella* species are endophytic in nature, rather than playing a role in symptom development. However, it has been noted that *R. victoriana* and *R. variigena* are isolated on a more frequent basis along with *G. quercinecans* and *B. goodwinii* prompting speculation about a possible beneficial symbiotic relationship with the bacteria responsible for the lesions and necrosis associated with AOD (unpublished data).

### The oak microbiome

After nearly 8 years of species identification, novel taxonomic classifications and rearrangements, the key bacterial species associated with AOD had been identified. The diversity of the species isolated and the fact that the majority of these species were novel species, or even genera, highlighted how little was known about the natural oak microbiome compared to that of symptomatic oak.

In an effort to remedy this, two studies were undertaken in parallel to determine the community structure of symptomatic oak versus healthy oak from five sites representing the known AOD distribution in England. A total of 15 trees, five healthy and ten symptomatic, were selected for sampling from the five sites. Bark panels (to the depth of the heartwood) were chiselled from the trees and later manually separated into outer bark, inner bark, sapwood and heartwood tissue. In addition to the healthy and symptomatic panels, non-symptomatic bark was sampled from symptomatic oak trees.

The first study used a metabarcoding technique, based on 454 amplicon sequencing of the 16S rRNA gene, to take into account those bacteria that cannot be cultured (Sapp et al. 2016). Precisely 88 tissue samples were processed from the bark panels. Following data analysis of the sequencing reads, the most abundant taxa (species level identification was not always possible, especially for the *Enterobacteriaceae*) belonged to the families *Pseudomonadaceae, Enterobacteriaceae, Halomonadaceae, Shewanellaceae* and *Acholeplasmataceae*. The communities differed at each site, with high phylogenetic diversity and thousands of different taxa found at two of the five sites. The diversity of bacteria from the different oak tissues was not consistent, although the bacteria from tissue containing *A. biguttatus* galleries were significantly different to those found in the heartwood. However, a relationship between the microbiome of the oak and its health status was detected.

Three phylotypes belonging to the genera *Pseudomonas, Halomonas* and *Shewanella* were found in 80% of healthy bark samples. A phylotype belonging to the *Enterobacteriaceae* showing a close phylogenetic relationship to *Brenneria goodwinii* was found in 80% of the advanced symptomatic tissues, suggesting this bacterial species plays a role in symptom development. The *Pseudomonas* phylotype was also found in 80% of the symptomatic tissue samples, giving cause to believe that this species is a natural member of the oak microbiome regardless of the health status of the tree. Because of the high variability at the different sampling sites, no definitive oak microbiome could be detected for symptomatic oak.

The second study was based on a conventional isolation and identification method of 4262 pieces of oak tissue from the bark panels used in the metabarcoding study (Denman et al. 2016). Identification of the isolated bacteria was by either *gyrB* or 16S rRNA gene sequencing and comparison with the NCBI Blast database. It was observed that a significantly higher proportion of bacteria were isolated from symptomatic tissue (96%) than from healthy tissue (65%) or non-symptomatic tissue from diseased trees (70%); and that the inner bark and sapwood yielded more bacterial isolates than the outer bark or heartwood. The main culturable bacterial taxa were found to belong to the following families: *Alcaligenaceae, Bacillaceae, Brucellaceae, Burkholderiaceae, Enterobacteriaceae, Enterococcaceae, Hyphomicrobiaceae, Moraxellaceae, Nocaridaceae, Paenibacilliaceae, Pseudomonadaceae, Rhizobiaceae* and *Xanthomonodaceae*. In contrast with the metabarcoding study, some of the tissue combinations from each site had specific bacterial communities while similar bacterial communities were noted across all sites for the remaining tissue combinations. This was credited to the highly similar bacterial communities isolated from symptomatic tissue across all sites.

Species belonging to the *Enterobacteriaceae*, specificially *Brenneria goodwinii, Erwinia billingiae, Gibbsiella quercinecans* and *Rahnella victoriana*, and *Pseudomonadaceae* (particularly a novel taxon most similar to *P. fulva*) were the dominant members of the culturable microbiome of symptomatic oak tissue at all five sites. The ratio of these species differed at each site with *B. goodwinii* more abundant at sites where AOD was further established and *G. quercinecans* in greater numbers at sites where the disease had progressed to a lesser extent. The recurrent isolation of certain species (*B. goodwinii, G. quercinecans* and *R. victoriana*) from the necrotic lesions of symptomatic tissue suggests that they play a specific role in disease development, and that necrosis is not due to opportunistic microorganisms. Bacteria belonging to Gram positive families and also *Pseudomonas* species constituted the community of the healthy oak microbiome. The results from Denman et al. (2016) indicate that there is a difference between the culturable microbiomes of healthy and diseased oak, but that more work is needed to pinpoint the role of the bacteria in the disease.

A separate study was performed on 64 healthy *Q. robur* trees cores in a single woodland site in the UK with no evident symptoms of AOD, also by amplification of the 16S rRNA gene from the total DNA of 192 samples, to explore the bacterial composition in pedunculate oak (Meaden et al. 2016). Bioinformatic analysis of the resulting sequences identified the bacteria to the class level only, with alphaproteobacteria being the most abundant class observed, followed by thermoleophilia and betaproteobacteria. Additionally, this study compared tree size to species richness and found a slight decline in species diversity as trees age, when observed OTUs (operational taxonomic units) were used as a measure of diversity. This could be attributed to differences in the chemical and physiological state of the host tissue. No *Brenneria* species were identified from any of the samples from these healthy trees, again suggesting that *B. goodwinii* could play an important role in AOD in Britain.

### Identification

The diversity of species associated with AOD (from *Enterobacteriaceae* to *Pseudomonadaceae*) determined by the taxonomic and microbiome studies, suggest a polymicrobial cause of disease. This has highlighted a need for the rapid and reliable identification of the key species involved in symptom expression. As the most frequently isolated species are all Gram-negative rods, with the majority belonging to the *Enterobacteriaceae*, identification based solely on phenotype is usually inaccurate. Sequencing of the 16S rRNA gene will yield an identity at the genus level, but below this (species or subspecies level) is not feasible due to the highly conserved nature of the gene between related members of enterobacteria. As previously mentioned, protein-encoding genes, particularly *gyrB* and *rpoB*, provide a higher resolution of discrimination for the *Enterobacteriaceae*. However, identification based on sequencing is laborious, time-consuming and costly.

A study was carried out aimed at differentiating between *G. quercinecans* and *B. goodwinii* targeting the 16S–23S internal transcribed spacer region 1 (ITS1) in a DNA fingerprinting method (Doonan et al. 2014). Primers designed for environmental bacterial communities were used to amplify the ITS1 region for 34 isolates from symptomatic oak. The resulting amplification products were separated by both agarose gel electrophoresis and polyacrylamide gel electrophoresis (PAGE). It was found that 3% agarose gels yielded some variable results in banding patterns and was deemed not sensitive enough for visualisation of the amplified fragments. However, polyacrylamide gel electrophoresis successfully separated the ITS1 amplified fragments which could be visualised after staining with SYBR Gold. The amplicons were scored according to size, converted to a binary output based on presence/absence and a distance-based tree constructed. Using the ITS1 profiles of the 34 isolates, they could be separated into two lineages corresponding to *G. quercinecans* and *B. goodwinii*. Although more cost-effective, the ITS1 fingerprinting method is no less labour intensive than sequencing and may be subject to bias based on the amplification rate of the different copies of the ITS1 region.

The decreasing costs of real-time PCR and next generation sequencing have opened new avenues for the development of rapid identification methods. A real-time PCR assay, based on species-specific *gyrB* sequence targets, was developed by researchers at Forest Research for rapid identification of *B. goodwinii* (Plummer et al. 2013). The assay was found to exclusively amplify *B. goodwinii* at an annealing temperature of 65 °C, whereas no probe reaction was observed for other *Brenneria* species, or species of *Gibbsiella, Rahnella, Lonsdalea* or *Serratia*. Tests to determine the assay’s sensitivity must still be performed. More recently, a relatively new technique called high resolution melt (HRM) analysis was investigated for rapid identification of four frequently isolated bacterial species from symptomatic oak in Britain (Brady et al. 2016). HRM analysis is a real-time PCR technique which can detect single nucleotide polymorphisms (SNPs) in short stretches of DNA without having to sequence the resulting amplicon. Amplification takes place in the presence of an intercalating dye which only fluoresces when bound to double-stranded DNA. A melting step, where the temperature increases incrementally until 95 °C is reached, follows amplification of the target region with the loss of fluorescence measured at each cycle. The rate of lost fluorescence corresponds to the G + C content of the amplified region generating a unique melt curve for each strain meaning that samples can be differentiated by a single base pair.

Primers targeting a heterogenous region of the *atpD* gene were used to amplify reference strains of *G. quercinecans, B. goodwinii. B. roseae* ssp. *roseae* and *L. quercina* ssp. *britannica* as well as their closest phylogenetic relatives. The melt curves generated for each strain were converted to scatter plots and principal component analysis performed. It was observed that the HRM technique could successfully cluster isolates according to identity and even differentiate between subspecies of *B. roseae* and *L. quercina*. HRM analysis has demonstrated great promise for a single identification assay which can rapidly identify multiple species associated with AOD. However, the current technique has only been tested on pure cultures and, like the ITS1 fingerprinting technique, is still reliant on the isolation of the bacteria from symptomatic tissue.

Due to the large number of tissue samples processed annually at Forest Research (the research agency of the United Kingdom Forestry Commission), ideally the rapid technique should directly target symptomatic tissue or fluid exudate bypassing the bacterial isolation and culturing steps. Work is currently underway to streamline the HRM technique to achieve this, and also to expand the identification of species to include *Rahnella* and *Pseudomonas* and to identify the bacteria from mixed cultures.

## Conclusions and future work

Investigations into the bacterial community of symptomatic oak have yielded a diverse range of novel genera and species, and have also greatly affected the taxonomic composition of the family *Enterobacteriaceae*. With the data yielded by the taxonomic and microbiome studies, it is now much clearer which organisms constitute the bacterial populations of symptomatic and non-symptomatic oak. It appears that *G. quercinecans* and *B. goodwinii* are the most frequently occurring species isolated from symptomatic oak. However, *R. victoriana, R. variigena* and the unclassified ‘*Pseudomonas fulva*-like’ species may play a secondary role in decline development as they are also isolated in relatively high numbers. As these species were only described in the past few years, there is little known regarding their origin, pathogenicity, biology or genetics. Consequently, this has opened up new avenues of research into AOD.

Ongoing progress in next generation sequencing techniques means the cost of whole genome sequencing has decreased drastically over the past 10 years. It is becoming commonplace for the whole genome sequence of the type strain of novel species to be performed and used in the polyphasic approach to species circumscription. Indeed, it has been suggested that the whole genome sequence of the type strain should accompany novel species descriptions (Rosselló-Móra and Amann 2015). Several different parameters are already in use to calculate the whole genome relatedness for the purpose of novel species circumscription, including ANI (average nucleotide identity), dDDH (digital DNA–DNA hybridization) and MUM (maximal unique matches). It is likely that any future novel species isolated from symptomatic oak will incorporate whole genome sequences, possibly simplifying the process and improving resolution of taxonomic issues.

In 2012, a large AOD consortium was established to focus research into the areas of pathogenicity, entomology, genomics and modelling. Researchers from several institutions are investigating factors which contribute to symptom development, such as predisposition of tree, environmental factors (e.g. topography, soil, pollutants, microclimate etc.), pests and pathogens and the host (metabolism, root health, genetics). This multi-disciplinary holistic approach should improve our understanding of oak health in general and lead to management of AOD, safeguarding the future of oak in Britain.
